# A Convenient, Rapid, Sensitive, and Reliable Spectrophotometric Assay for Adenylate Kinase Activity

**DOI:** 10.3390/molecules24040663

**Published:** 2019-02-13

**Authors:** Kai Song, Yejing Wang, Yu Li, Chaoxiang Ding, Rui Cai, Gang Tao, Ping Zhao, Qingyou Xia, Huawei He

**Affiliations:** 1 Biological Science Research Center, Southwest University, Beibei, Chongqing 400715, China; Kaisong@email.swu.edu.cn (K.S.); liyu315@swu.edu.cn (Y.L.); ding7197767@email.swu.edu.cn (C.D.); taogang@email.swu.edu.cn (G.T.); zhaop@swu.edu.cn (P.Z.); xiaqy@swu.edu.cn (Q.X.); 2 State Key Laboratory of Silkworm Genome Biology, College of Biotechnology, Southwest University, Beibei, Chongqing 400715, China; cairui0330@email.swu.edu.cn; 3 Chongqing Key Laboratory of Sericultural Science, Chongqing Engineering and Technology Research Center for Novel Silk Materials, Southwest University, Beibei, Chongqing 400715, China

**Keywords:** enzymatic activity assay, adenylate kinase, spectrophotometry, orthogonal experiment, bromothymol blue

## Abstract

Enzymatic activity assays are essential and critical for the study of enzyme kinetics. Adenylate kinase (Adk) plays a fundamental role in cellular energy and nucleotide homeostasis. To date, assays based on different principles have been used for the determination of Adk activity. Here, we show a spectrophotometric analysis technique to determine Adk activity with bromothymol blue as a pH indicator. We analyzed the effects of substrates and the pH indicator on the assay using orthogonal design and then established the most optimal assay for Adk activity. Subsequently, we evaluated the thermostability of Adk and the inhibitory effect of KCl on Adk activity with this assay. Our results show that this assay is simple, rapid, and precise. It shows great potential as an alternative to the conventional Adk activity assay. Our results also suggest that orthogonal design is an effective approach, which is very suitable for the optimization of complex enzyme reaction conditions.

## 1. Introduction

Adenylate kinase (Adk; ATP:AMP phosphotransferase, EC 2.7.4.3), also known as myokinase, is a conserved phosphoryl transferase, which catalyzes the translocation of a phosphoryl group between nucleotides in the reversible reaction (AMP + Mg^2+^•ATP ↔ Mg^2+^•ADP + ADP) [1]. Adk is ubiquitous in different tissues of all living systems and plays a fundamental role in cellular energy and nucleotide homeostasis. The hydrogen bond between the adenine moiety and the backbone of Adk is critical for ATP selectivity and can help Adk recognize the correct substrates in the complex cellular environment [2]. Adk is involved in the regulation of cell differentiation, maturation, apoptosis, and oncogenesis. Adk mutations in humans cause a severe disease called reticular dysgenesis [3]. Adk is regarded as a potential target for medical diagnosis and treatment due to its close correlation with other diseases, such as aleukocytosis, hemolytic anemia, and primary ciliary dyskinesia [4]. To date, three methods have been proposed to determine Adk activity based on the detectable changes accompanied with this reaction, such as light absorption, acidity, or the coupled reaction products [5]. The manometric assay, established by Colowick and Kalckar [6], is used for the detection of Adk by measuring CO_2_ liberation from a bicarbonate buffer. The reaction catalyzed by Adk is coupled to hexokinase, which specifically catalyzes the transformation of the terminal phosphate from ADP to glucose. The overall reaction is as follows:

(1) $$\mathrm{ATP}+\mathrm{AMP}\overset{\mathrm{Adk}}{↔}\mathrm{ADP}+\mathrm{ADP}$$

(2) $$\mathrm{ADP}+\mathrm{glucose}\overset{\mathrm{hexokinase}}{\to }\mathrm{glucose}-6-P+\mathrm{AMP}+H^{+}$$

In the presence of excess hexokinase, the reaction rate is proportional to the Adk concentration. The forward direction reaction is defined as the formation of AMP and ATP from two ADPs, and the reverse direction is defined as the formation of two ADPs from AMP and ATP. Adk activity is conventionally measured in vitro by a spectrophotometric assay. For the forward direction reaction, Chiu et al. [7] have developed a modified assay of Oliver [8] to determine Adk activity by coupling the reaction to hexokinase and glucose-6-phosphate dehydrogenase in which the final product, NADPH, is measured spectrophotometrically at 340 nm. The overall reaction is as follows: H^+^

(3) $$\mathrm{ADP}+\mathrm{ADP}\overset{\mathrm{Adk}}{↔}\mathrm{ATP}+\mathrm{AMP}$$

(4) $$\mathrm{ATP}+\mathrm{glucose}\overset{\mathrm{hexokinase}}{\to }\mathrm{glucose}-6-P+\mathrm{ADP}$$

(5) $$\mathrm{glucose}-6-P+\mathrm{NADP}\overset{\begin{array}{c} \mathrm{glucose}-6-\mathrm{phosphate}\;\\ \mathrm{dehydrogenase} \end{array}}{\to }6-\mathrm{phosphogluconic}\;\mathrm{acid}+\mathrm{NADPH}$$

Adk activity is also measured in the reverse direction by coupling the reaction to pyruvate kinase and lactate dehydrogenase and measuring the oxidation of NADH at 340 nm [5]. The principle of the assay is as follows:

(6) $$\mathrm{ATP}+\mathrm{AMP}\overset{\mathrm{Adk}}{↔}\mathrm{ADP}+\mathrm{ADP}$$

(7) $$\mathrm{Phosphoenolpyruvate}+\mathrm{ADP}\overset{\mathrm{Pyruvate}\;\mathrm{kinase}}{\to }\mathrm{ATP}+\mathrm{Pyruvate}$$

(8) $$\mathrm{Pyruvate}+\mathrm{NADH}+H+\overset{\mathrm{Lactic}\;\mathrm{dehydrogenase}}{\to }\mathrm{Lactate}+{\mathrm{NAD}}^{+}$$

These assays have been used to determine Adk activity for the past decades. However, some disadvantages are also obvious for these assays. Firstly, these assays are time-consuming, multistep processes that require the assistance of other enzymes and are easily subject to errors at each step. Secondly, it is difficult to study the effects of activators and inhibitors on Adk activity with the aid of other enzymes. Finally, the real initial rate of Adk reaction cannot be determined accurately [9]. Therefore, it is necessary to develop a more convenient and accurate assay for Adk activity in vitro.

Acid–base indicators are usually applied in enzymatic assay for their extraordinary sensitivity to pH change. In 2002, Yu et al. [10] established an arginine kinase activity assay based on the light absorption of a complex acid–base indicator consisting of thymol blue and cresol red. In the reaction catalyzed by arginine kinase, the produced protons resulted in a decrease in pH of the reaction mixture, thus reducing the absorbance of the mixed indicator in the solution at 575 nm. The arginine kinase activity could be determined according to the change of the absorbance at 575 nm. In the same way, Dhale et al. developed a rapid and sensitive assay to measure l-asparaginase activity with methyl red as an indicator [11]. Bromothymol blue is an excellent indicator as it forms a highly conjugated structure while deprotonated in alkaline solution, resulting in an obvious color change from yellow to blue and the corresponding absorbance change [12].

Enzyme activity is typically influenced by many factors. The traditional method is to do a multifactor analysis that tests all possible combinations of the different factors. However, this takes up a lot of time and resources as the number of full factorial experiments is very large. As an alternative, the orthogonal design method has been proposed and established. The orthogonal experimental design [13] is a multifactor experiment design assay. It selects representative samples from a full factorial assay in a way that the samples are distributed uniformly within the test range, thus representing the overall situation. Therefore, it is highly efficient for the arrangement of multifactor experiments with optimal combination levels. The orthogonal design has three advantages: (1) The number of tests required to complete the experiment is relatively small. (2) The data points are evenly distributed. (3) The test results can be analyzed by mathematical calculation (e.g., range analysis and variance analysis), which is particularly useful to quantify the results.

In this study, we developed a one-step assay for Adk activity. It is based on proton generation after the addition of ATP and AMP as the substrates, which can be measured spectrophotometrically at 614 nm using bromothymol blue as a pH indicator. We investigated four factors affecting Adk activity—ATP, AMP, bromothymol blue, and glycine–NaOH buffer—at three levels and determined the best combination for Adk activity assay by an orthogonal experimental design. Finally, we evaluated the thermostability of Adk and the inhibitory effect of KCl on Adk activity with this assay. Our results suggest that this assay is simple, precise, less expensive, and a potential alternative to the conventional enzymes-coupled assay extensively used in clinical and research laboratories.

## 2. Results

**Figure 1:**
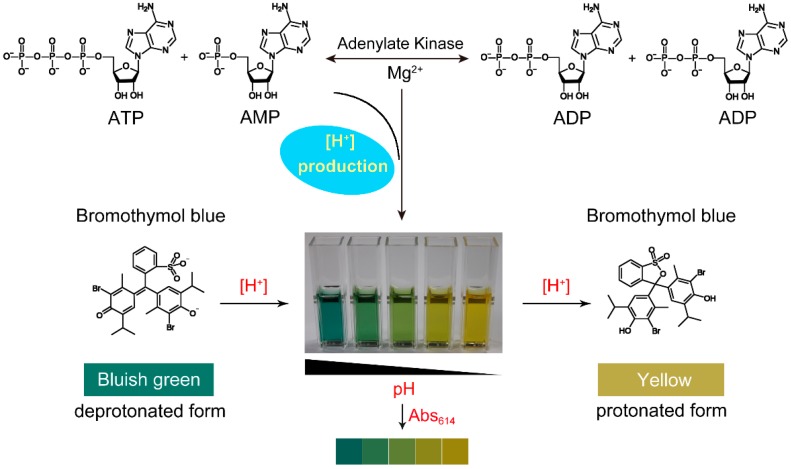
The principle of the spectrophotometric assay for adenylate kinase (Adk) activity.

In this study, Adk activity was determined by a direct and continuous spectrophotometric technique without coupled enzymes. In the enzymatic reaction catalyzed by Adk, the formation of two ADPs from ATP and AMP is accompanied by the generation of hydrogen ions. Bromothymol blue is an excellent acid–base indicator as it forms a highly conjugated structure while protonated in acid solution, resulting in an obvious color change from blue to yellow. The absorbance of bromothymol blue at 614 nm is associated with the hydrogen ion concentration in solution. Thus, Adk activity can be monitored in real time by the absorbance of bromothymol blue at 614 nm in solution, which can be detected by a sensitive spectrophotometer. The principle of this assay is illustrated in Figure 1. 

The effects of substrates and the pH indicator on the assay were analyzed using an orthogonal design, which is key to establishing the most optimal assay for Adk activity. The factors and levels affecting Adk activity assay are shown in Table 1.

### 2.1. The Maximum Absorption Wavelength of Reaction Mixture

**Figure 2:**
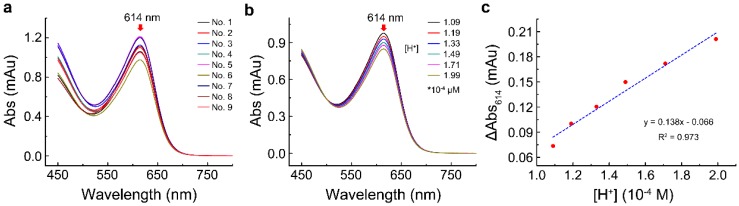
The maximum absorption wavelength of the reaction mixture and its relationship with the hydrogen ion concentration. (**a**) The absorption spectra of nine different combinations in the presence of 5 mM MgAC_2_; (**b**) effect of hydrogen ion concentration on the absorption of the reaction system; (**c**) the correlation of the absorbance change of bromothymol blue at 614 nm with hydrogen ion concentration.

A set of nine tests designed by orthogonal experiment is shown in Table 2. The absorption spectrum of each set was scanned from 450 to 800 nm in the presence of 5 mM MgAC_2_. All spectra showed the same maximum absorption located at 614 nm without shift, as shown in Figure 2a.

### 2.2. Optimization of Adk Activity Assay

The effects of ATP, AMP, bromothymol blue, and glycine–NaOH buffer on the Adk activity were analyzed, as shown in Table 2. The significance of these factors on the assay was determined by the range (R) value listed in Table 2. The results showed that the order of R value was RC ˃ RB ˃ RD ˃ RA. Hence, the significance order of these factors on the assay was C ˃ B ˃ D ˃ A, namely, bromothymol blue ˃ AMP ˃ glycine–NaOH buffer ˃ ATP. Similarly, the significance of different levels on the assay was determined by the ti value listed in Table 2. The results showed that the orders were t1 ˃ t3 ˃ t2 for factor A, t1 ˃ t2 ˃ t3 for factor B, t1 ˃ t2 ˃ t3 for factor C, and t1 ˃ t2 ˃ t3 for factor D. Thus, the most optimal combination for Adk activity assay was A1B1C1D1, which was composed of 2 mM ATP, 1 mM AMP, 0.093 mM bromothymol blue, and 0.1 mM glycine–NaOH buffer. 

Next, we conducted a full factorial design for two primary factors of bromothymol blue and AMP at three levels to further optimize the assay. The design matrix and parameters are listed in Table 3. The results showed that AB3C1D had the most significant absorbance change at 614 nm among all the tested conditions (Table 4), indicating AB3C1D (2 mM ATP, 0.6 mM AMP, 0.093 mM bromothymol blue, and 0.1 mM glycine-NaOH buffer) was the most optimal reaction condition for the Adk activity assay.

### 2.3. Effect of H^+^ on the Absorbance of Bromothymol Blue

To determine the sensitivity of bromothymol blue on the Adk activity assay, we measured the response of bromothymol blue to hydrogen ion under the most optimal condition, AB3C1D. The absorbance spectrum of bromothymol blue was scanned from 450 to 800 nm in the presence of various concentrations of HCl. The results showed that the absorbance of bromothymol blue at 614 nm gradually declined with the increase in hydrogen ion concentration (Figure 2b). The absorbance change can be linearly fitted as the function of hydrogen ion concentration with the following equation (Figure 2c):

y = 0.138 ∗ x − 0.066

The results suggested that the absorbance of bromothymol blue at 614 nm had a good response to pH change in the assay, and the absorbance change was positively correlated with the hydrogen ion concentration.

### 2.4. Effect of Adk Contents on the Reaction Velocity

**Figure 3:**
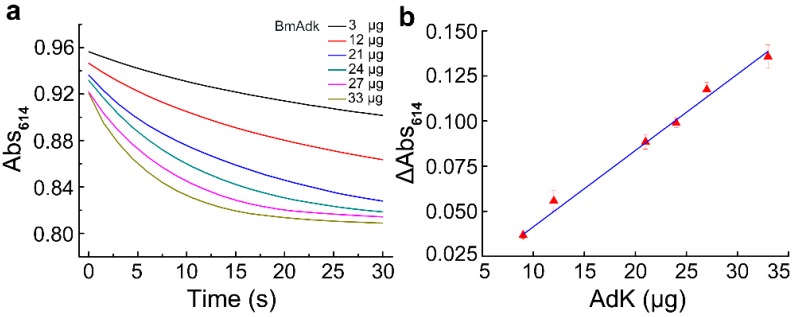
The effect of Adk contents on the assay. (**a**) Effect of different Adk contents on the assay; (**b**) the correlation of the absorbance change of bromothymol blue at 614 nm with different Adk contents.

The effect of Adk contents on the reaction velocity was determined as described in the Materials and Methods section. The results showed that, with the increase in Adk contents, the reaction velocity increased, and the reaction time required to reach equilibrium shortened (Figure 3a). In the first 5 s, the absorbance of bromothymol blue at 614 nm (Abs_614_) declined linearly with time; thus, the slope of the reaction in the first 5 s was defined as the initial reaction velocity. The plot in Figure 3b shows that the absorbance change of bromothymol blue at 614 nm could be linearly fitted as the function of Adk contents.

### 2.5. Effect of Temperature and KCl on Adk Activity

**Figure 4:**
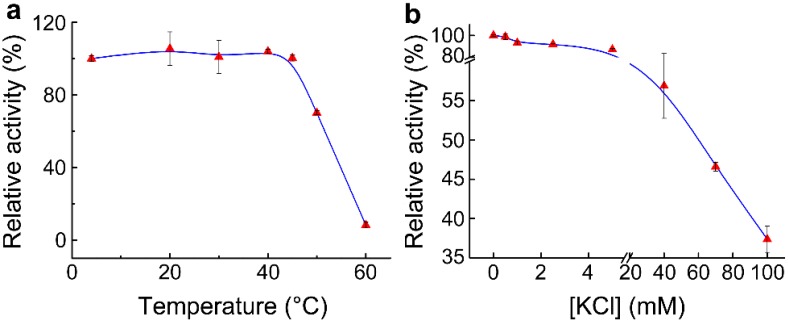
The thermostability of Adk and the inhibition of KCl on Adk activity. (**a**) Effect of temperature on Adk activity; (**b**) effect of KCl concentration on Adk activity.

The effect of temperature on Adk activity was investigated to characterize the thermostability of Adk. The results showed that Adk activity was almost unaffected under 45 °C. However, when the temperature was increased to 60 °C, Adk quickly lost its activity (Figure 4a). Adk from the muscle has a half-life of 30 min in 0.1 N hydrochloric acid at 100 °C [14]. Our results indicate that the thermostability of Adk from *Bombyx mori* (BmAdk) is much lower than that of Adk from the muscle.

Allan Hough et al. proved that KCl can almost completely inhibit myokinase activity [15]. Here, the effect of KCl on Adk activity was assessed with the assay. The results showed that low concentration of KCl (<5 mM) had a slight inhibitory effect on Adk activity. With the increase in KCl concentration, the inhibitory effect of KCl on Adk activity became more and more obvious. About 70 mM KCl resulted in 50% loss of Adk activity (Figure 4b). Compared with the “three-minute” method [15], the inhibitory effect of KCl on Adk activity could be assessed more easily with our developed assay.

## 3. Materials and Methods

### 3.1. Chemicals and Materials

ATP and AMP were purchased from Aladdin (Shanghai, China) in the form of sodium salt. Magnesium acetate was from Sigma (St. Louis, MO, USA). Bromothymol blue sodium salt, glycine, and other reagents all came from Sangon Biotech Corp. (Shanghai, China). Plastic cuvettes were purchased from Centome Corp. (Chengdu, China).

### 3.2. Adk Preparation and Concentration Determination

The DNA fragment encoding Adk was obtained from the cDNA library by PCR from the midgut of *Bombryx mori* strain Dazao using primer sets (5′-ATGGCACCGGCCGCTGC-3′ and 5′-TTACAAA GCAGACCGTGCTCTGCTG-3′). The amplification product was gel-purified, recovered, and inserted into plasmid vectors pSKB2. The bacterial transformants containing error-free inserts were identified. Adk was expressed in *Escherichia coli* BL21(DE3) and purified by Ni-NTA affinity chromatography (GE Healthcare, Chicago, IL, USA). The fused polyhistidine tag was cleaved by Prescission protease (GE Healthcare, USA) and removed as described by Liu et al. [16]. Protein concentration was determined using the extinction coefficient of 12,950 M^−1^·L·cm^−1^ at 280 nm on a NanoDrop 2000C spectrophotometer (Thermo Fisher, Waltham, MA, USA).

### 3.3. Adk Activity Assay

ATP and AMP were used as the substrates in the assay. The reaction mixture (1 mL) was composed of 2 mM ATP, 0.6 mM AMP, 0.1 mM glycine–NaOH (pH 9.0), 0.093 mM bromothymol blue, and 5 mM MgAC_2_. The initial absorbance of the freshly prepared reaction mixture at 614 nm was adjusted to 1.05 with approximately 0.5 M NaOH so that the absorbance of the mixture would not be changed after the addition of Adk. The purified Adk was exchanged into buffer A (20 mM Tris-HCl, pH 7.6, 150 mM NaCl, 5% glycerol, and 0.1 mM dithiothreitol (DTT)) via gel filtration. The reaction was triggered by adding 15 μg Adk into the mixture. The reaction velocity was defined as the slope of the absorbance change of bromothymol blue at 614 nm in the initial 30 s, which was recorded on a DU 800 nucleic acid/protein analyzer (BeckmanCoulter, Brea, CA, USA) using a 1-cm light path plastic cuvette. For the control reaction, Adk was replaced with buffer A. All measurements were carried out at 25 °C. Each test was replicated at least three times.

### 3.4. Orthogonal Design

The concentration of ATP, AMP, bromothymol blue, and glycine–NaOH buffer is vital for Adk activity assay. Consequently, these factors were considered in the orthogonal design to screen the most optimal conditions for Adk activity. Assuming there were three levels for each factor, and the interaction among the factors were not taken into account, the orthogonal experiment of four factors at three levels was designed.

## 4. Discussion

Adk plays a crucial role in maintaining a balance of cellular energy and nucleic acid metabolism. Human Adk isoenzymes specifically expressed in organs are regarded as important indicators of organ dysfunction [17] or differentiation stages [18]. The Adk activity assays that have been developed so far are largely dependent on the coupled secondary enzymes hexokinase and glucose-6-phosphate dehydrogenase or adenosine monophosphate deaminase [19]. In addition, the different conditions between the coupled enzymes and Adk affect the application of these assays [14]. Therefore, developing a convenient, rapid, sensitive, reliable, and economic assay for Adk activity is of great significance.

Here, we developed a spectrophotometric assay for Adk activity using bromothymol blue as a pH indicator without coupled enzymes. The effective range of bromothymol blue is pH 6.0–7.6. Adk is optimally active at pH 7.6 [14]. Hence, Adk was dissolved in pH 7.6 buffer to keep it active. The correspondence of pH and the absorbance of the system are listed in Table 5. Here, we set the initial absorbance of 1.05 as the beginning of the reaction, which represented pH 6.8 of the reaction system. When 5 μL buffer was added into the system (995 μL), there was almost no change in the pH of the system. At the same time, the assay system could ensure bromothymol blue had a sensitive and stable response to pH change caused by Adk-catalyzed reaction.

The effects of ATP, AMP, bromothymol blue, and glycine–NaOH buffer on the Adk activity assay were determined by a rational orthogonal design. For a full factorial assay with four factors at three levels, the number of tests is up to 81 (3^4^). However, our rational orthogonal design greatly reduced the unnecessary experiments and effectively achieved significant results (Table 2). The orthogonal experimental design is a rational design method for multifactor experiment, which selects representative points from a full factorial assay to represent the overall situation. Therefore, it is highly efficient for the design of multifactor experiments with optimal combination levels. By means of the orthogonal design, we greatly reduced the number of required experiments and achieved significant results with the least number of experiments.

The optimal temperature for the growth of *Bombyx mori* is about 25 °C. Adk from *Bombyx mori* was relatively stable below 45 °C, implying its significance on the growth and development of *Bombyx mori*. The Adk structure suggests that the hydrophobic core packing is important for the stability and activity of Adk [20]. AMP has a strong inhibition effect on the reaction with ADP as the substrate, thus resulting in a rapid decrease in the reaction rate with time [6,19]. Here, we found that high concentration of AMP could inhibit Adk activity, which is in line with a previous report. Slater et al. [21] found that the inhibitory effect of AMP was easily observed even when excessive hexokinase and glucose were present. The inhibition was ascribed to the affinity of ADP to Adk, which is lower than that of AMP with Adk. However, in this study, the substrates were ATP and AMP. Therefore, the inhibition of AMP on Adk activity may be attributed to the noncompetitive inhibition of AMP with respect to ATP [22].

To summarize, we developed a simple and rapid assay to determine Adk activity with bromothymol blue as an indicator instead of coupled enzymes. The assays that have been developed so far require the assistance of other enzymes to convert the reaction product to other detectable signals; thus, they consist of multiple reaction steps and are discontinuous. The assays are not accurate as they are easily subject to errors at each step. However, this new assay only relies on the protons produced in the reaction and the corresponding absorbance changes of bromothymol blue in the reaction solution. Small changes in pH can lead to significant changes in the absorbance of bromothymol blue at 614 nm. It does not need the assistance of any other enzyme. Bromothymol blue is less expensive than the coupled enzymes, and the assay can be done in just one step, thus reducing the chance of errors and improving reliability. Compared with the existing assays, it is more simple, sensitive, and precise. In addition, it can be applied to research the activation or inhibition of Adk as it is continuous. However, although this assay is simple and precise, the reaction substrate must be prepared freshly as carbon dioxide in the air may interfere with the assay. ATP spontaneously and slowly hydrolyzes in solution, which can also cause an interference on the assay. Nevertheless, it can be a good alternative to the conventional enzymes-coupled Adk activity assay extensively used in clinical and research laboratories.

## Figures and Tables

**Table 1:** Factors and levels affecting Adk activity assay.

Level	A	B	C	D
	ATP (mM)	AMP (mM)	Bromothymol Blue (mM)	Glycine–NaOH (mM)
1	2.0	1.0	0.0930	0.1
2	2.5	1.5	0.1084	0.3
3	3.0	2.0	0.1238	0.5

**Table 2:** Orthogonal array (9, 3^4^) for the analysis of the effects of ATP, AMP, bromothymol blue, and glycine–NaOH buffer on the Adk activity assay.

No.	Combination	Factor				ΔAbs ^a^ (0–30 s)
		A	B	C	D	
1	A_1_B_1_C_1_D_1_	2.0	1.0	0.0930	0.1	0.0914 ± 0.0020
2	A_1_B_2_C_2_D_2_	2.0	1.5	0.1084	0.3	0.0432 ± 0.0045
3	A_1_B_3_C_3_D_3_	2.0	2.0	0.1238	0.5	0.0112 ± 0.0017
4	A_2_B_1_C_2_D_3_	2.5	1.0	0.1084	0.5	0.0404 ± 0.0017
5	A_2_B_2_C_3_D_1_	2.5	1.5	0.1238	0.1	0.0221 ± 0.0020
6	A_2_B_3_C_1_D_2_	2.5	2.0	0.0930	0.3	0.0606 ± 0.0022
7	A_3_B_1_C_3_D_2_	3.0	1.0	0.1238	0.3	0.0341 ± 0.0009
8	A_3_B_2_C_1_D_3_	3.0	1.5	0.0930	0.5	0.0705 ± 0.0049
9	A_3_B_3_C_2_D_1_	3.0	2.0	0.1084	0.1	0.0382 ± 0.0017
T_1_		0.1458	0.1659	0.2225	0.1517	
T_2_		0.1231	0.1358	0.1218	0.1379	
T_3_		0.1428	0.1100	0.0674	0.1221	
t_1_		0.0486	0.0553	0.0742	0.0506	
t_2_		0.0410	0.0453	0.0406	0.0460	
t_3_		0.0476	0.0367	0.0225	0.0407	
Range (R)		0.0076	0.0186	0.0517	0.0099	
Order		C ˃ B ˃ D ˃ A				
Optimal level		A_1_	B_1_	C_1_	D_1_	
Optimal combination		A_1_B_1_C_1_D_1_				

^a^ Arithmetic mean of the absorbance changes of bromothymol blue at 614 nm (0–30 s) of three independent tests at each level under the same factor. Ti (T1, T2, T3) is the sum of the recorded absorbance changes (ΔAbs) at the same level and under the same factor, and ti (t1, t2, t3) is the arithmetic mean of Ti. The mean of ti represents the influence of different levels under the same factor on the absorbance of bromothymol blue. Range (R) is the difference between the maximum and the minimum of ti, indicating the effect of each factor on the absorbance of bromothymol blue. The greater the R value, the greater is the influence of this factor on the absorbance of bromothymol blue at 614 nm.

**Table 3:** Factors and levels affecting Adk activity assay with the constants of A and D.

Level	A	B	C	D
	ATP (mM)	AMP (mM)	Bomothymol Blue (mM)	Glycine–NaOH (mM)
1	2.0	1.0	0.0930	0.1
2	2.0	0.8	0.0775	0.1
3	2.0	0.6	0.0620	0.1

**Table 4:** A full factorial design for the analysis of the effects of AMP, bromothymol blue on the Adk activity assay with the constants of A and D.

Run	Combination	Factor				ΔAbs ^b^ (0–30 s)
		A	B	C	D	
1	AB_1_C_1_D	2.0	1.0	0.0930	0.1	0.0914 ± 0.0020
2	AB_1_C_2_D	2.0	1.0	0.0775	0.1	0.0926 ± 0.0030
3	AB_1_C_3_D	2.0	1.0	0.0620	0.1	0.0764 ± 0.0030
4	AB_2_C_1_D	2.0	0.8	0.0930	0.1	0.0914 ± 0.0020
5	AB_2_C_2_D	2.0	0.8	0.0775	0.1	0.0930 ± 0.0018
6	AB_2_C_3_D	2.0	0.8	0.0620	0.1	0.0680 ± 0.0041
7	AB_3_C_1_D	2.0	0.6	0.0930	0.1	0.0983 ± 0.0028
8	AB_3_C_2_D	2.0	0.6	0.0775	0.1	0.0850 ± 0.0054
9	AB_3_C_3_D	2.0	0.6	0.0620	0.1	0.0604 ± 0.0001

^b^ Arithmetic mean of the absorbance changes of bromothymol blue at 614 nm (0–30 s) of three independent tests at each level under the same factor.

**Table 5:** Correspondence between pH and the absorbance of bromothymol blue at 614 nm.

pH	6.47	6.54	6.73	6.79	6.85	7.07	7.21	7.28
Abs	0.8145	0.9197	0.9438	1.0375	1.0847	1.2385	1.3850	1.4188

## Abbreviations

Adk	Adenylate kinase
ATP	adenosine 5′-triphosphates
ADP	adenosine 5′-diphosphates
AMP	adenosine 5′-monophosphate
NADPH	reduced nicotinamide adenine dinucleotide phosphate
Abs	absorbance
